# Epidemiological and clinical characteristics of hospitalized unintentional injuries among children in central China from 2017–2023

**DOI:** 10.3389/fped.2024.1381287

**Published:** 2024-05-23

**Authors:** Meng Wang, Yibing Cheng, Haijun Wang, Li Lin, Yuelin Shen

**Affiliations:** ^1^ Emergency Department, Children’s Hospital Affiliated to Zhengzhou University, Henan Children’s Hospital, Zhengzhou Children’s Hospital, Henan Province Engineering Research Center of Diagnosis and Treatment of Pediatric Infection and Critical Care, Zhengzhou, China; ^2^ Respiratory Department II, National Clinical Research Center for Respiratory Diseases, Beijing Children’s Hospital, Capital Medical University, National Center for Children’s Health, Beijing, China; ^3^ Respiratory Department, Children’s Hospital Affiliated to Zhengzhou University, Henan Children’s Hospital, Zhengzhou Children’s Hospital, Henan Province Engineering Research Center of Diagnosis and Treatment of Pediatric Infection and Critical Care, Zhengzhou, China

**Keywords:** unintentional injury, external cause, trend change, children, central China

## Abstract

**Objectives:**

To examine the epidemiological and clinical characteristics of hospitalized unintentional injuries among children in Central China and theoretically propose preventive and control measures.

**Methods:**

We conducted a retrospective study of children aged 0–18 years with unintentional injuries who were admitted to a tertiary hospital in Central China from January 2017 to December 2023. We examined various aspects of the unintentional injuries, including age, gender, urban-rural distribution, external causes, trends, location of injury, cost, and length of stay.

**Results:**

A total of 20,166 children with hospitalized unintentional injuries were enrolled. The median age with IQR was 2.8 (1.6, 5.1) years, with majority of the patients (57.0%) were aged 1–3 years, while the fewest were aged 11–18 years. The male-to-female ratio was 1.8:1, and the urban-to-rural ratio was 1.1:1. The most common external causes were foreign bodies (41.7%), exposure to inanimate mechanical forces (25.1%), and falls (22.1%). The most frequently injured body parts were head (72.5%). The total number of unintentional injuries exhibited an increasing trend from 2017–2022, and a decreasing trend from 2022–2023. The urban-rural distribution reversed after 2020. The overall hospitalization cost was 20,810,870.4 USD, with an median cost of 758.7 (556.4, 1,186.2) USD per person.

**Conclusion:**

Unintentional injuries imposed a heavy burden on society and families. However, the number of cases and the urban-rural distribution showed significant trend changes from 2017–2023. The external causes varied by age group, gender, and region, while prevention and control measures should be developed accordingly.

## Introduction

Injuries refer to tissue damage caused by energy exchange of exercise, heat, chemistry, electricity, or radiation exceeding the tolerance level of the body's tissues, as well as hypoxia caused by suffocation. Currently, injury has become one of the major causes of death and disability among children and adolescents worldwide (1). Globally, there were nearly 2,300 children and adolescents died from injuries every day (2). According to the intention of injury, it can be divided into intentional injury and unintentional injury. Unintentional injuries, which occur without predetermined intent, include road traffic injuries, burns, drowning, poisoning, falls, suffocation, and sports-related injuries, etc. (3, 4).They account for about 80% of all injury deaths worldwide with a disproportionate burden on low- and middle-income countries where access to health care is limited (5)_._ In China, injuries cause approximately 65,000 deaths annually among children under 14 years of age, making them the leading cause of mortality in this age group (6, 7). In 2013, China ranked fifth in the world in the number of deaths due to unintentional injuries among children under 5 years of age (8) Road traffic injuries and drowning are the leading causes of child deaths worldwide due to unintentional injuries (9). However, the occurrence and patterns of unintentional injuries vary significantly across regions and populations, depending on their geographic environment and economic status (10–12). Most of the reports on unintentional injuries in China have focused on the southwestern (13) and southern regions (14), while Central China, which had a large population (223 million) by the end of 2017, remains largely understudied. In this study, we conducted a multi-perspective analysis of hospitalized unintentional injury among children aged 0–18 years in Central China, to explore the temporal trends, and to recommend some preventive strategies and measures based on our findings.

## Methods

### Subjects

We conducted a retrospective study of consecutive children with unintentional injuries aged under 18 years who were admitted to the Children's Hospital Affiliated to Zhengzhou University between January 2017 and December 2023. If a child was hospitalized more than once, only the first admission was counted. The enrolled patients were divided into five age groups: <1 year, 1–<4 years, 4–<7 years, 7–<11 years, and 11–18 years. This study was approved by the Ethics Committee of Children's Hospital Affiliated to Zhengzhou University, Zhengzhou, China (2024-K-007). Informed consent was obtained from all individual participants included in the study.

### Clinical information

We retrieved the hospitalization information of all patients with unintentional injuries through the Hospital Information System (HIS). We collected the demographic information of the patients, including age, sex, and region (urban/rural), as well as the clinical data, such as the mechanism and location of injury, the length of hospital stay, and the hospitalization cost.

The external cause codes were classified according to the WHO International Classification of Diseases-10 (ICD-10). Codes used in this study were as follows: transport accidents (V01–V09), falls (W00–W19), exposure to inanimate mechanical forces (EIMF, W20–43, W46–49), exposure to animate mechanical forces (EAMF, W50–W64), drowning (W65–W74), foreign body (W44–W45), poisoning (X40–X49), burn and scald (X00–X19).

### Statistical analysis

Statistical analyses were performed using SPSS software (version 22.0; SPSS, Chicago, IL, USA). The continuous data were expressed as mean ± standard deviation (SD) or median (interquartile range, IQR) according to the distribution unless otherwise specified. The categorical data represent as numbers (percentages). Comparisons according to group assignment were made with a Kruskal-Wallis test followed by nonparametric Bonferroni multiple comparison tests for continuous variables and χ^2^ test with Yates correction was used for categorical variables. A *p* value of less than 0.05 (two-tailed) was considered statistically significant.

## Results

### Demographic data

This study recruited a total of 20,166 pediatric patients, all of whom were Han Chinese from Central China. Among these patients, 13,069 (64.8%) were males, and 7,097 (35.2%) were females, resulting in a male-to-female ratio of 1.8:1. The median age of all the patients was 2.8 (1.6, 5.1) years old. The majority of the patients (11,497 cases, 57.0%) fell within the 1–4 years age group, while the fewest cases (259 cases, 1.3%) were observed in the 11–18 years age group. The overall urban-to-rural ratio was 1.1:1 (Table 1).

**Table 1:** The distribution of causes of unintentional injuries in central China, 2017–2023.

	2017	2018	2019	2020	2021	2022	2023	Total
Total number	1,870 (9.2%)	2,416 (12.0%)	2,663 (13.2%)	3,263 (16.2%)	3,631 (18.0%)	3,081 (15.3%)	3,242 (16.1%)	20,166 (100%)
Age, y	2.0 (1.0, 3.2)	2.8 (1.3, 3.5)	2.3 (1.5, 4.3)	2.6 (1.5, 4.7)	3.1 (1.8, 5.2)	3.8 (2.0, 5.8)	4.4 (2.6, 6.7)	2.8 (1.6, 5.1)
Sex								
Male	1,162 (8.9%)	1,534 (11.7%)	1,821 (13.9%)	2,300 (17.6%)	2,280 (17.5%)	1,927 (14.8%)	2,045 (15.6%)	13,069 (100%)
Female	708 (9.9%)	883 (12.4%)	847 (11.9%)	963 (13.6%)	1,351 (19.1%)	1,148 (16.2%)	1,197 (16.9%)	7,097 (100%)
Location								
Urban	456 (4.4%)	838 (8.2%)	1,096 (10.7%)	1,709 (16.6%)	2,138 (20.7%)	2,157 (20.9%)	1,911 (18.5%)	10,305 (100%)
Rural	1,414 (14.3%)	1,578 (16.0%)	1,567 (15.9%)	1,554 (15.8%)	1,493 (15.1%)	924 (9.4%)	1,331 (13.5%)	9,861 (100%)
Causes of injuries								
Foreign body	1,242 (14.8%)	1,704 (20.2%)	1,496 (17.7%)	1,340 (15.9%)	1,047 (12.5%)	761 (9.1%)	824 (9.8%)	8,414 (100%)
Falls	443 (9.9%)	477 (10.7%)	742 (16.6%)	642 (14.4%)	662 (14.9%)	623 (14.0%)	869 (19.5%)	4,458 (100%)
Transport accidents	39 (3.9%)	101 (9.9%)	116 (11.4%)	140 (13.8%)	215 (21.1%)	181 (17.8%)	225 (22.1%)	1,017 (100%)
Poisoning	62 (8.6%)	57 (7.9%)	118 (16.3%)	134 (18.5%)	134 (18.5%)	93 (12.9%)	125 (17.3%)	723 (100%)
Burn and scald	32 (12.9%)	30 (12.1%)	23 (9.3%)	63 (25.4%)	40 (16.1%)	29 (11.7%)	31 (12.5%)	248 (100%)
EIMF	22 (0.4%)	28 (0.5%)	132 (2.6%)	911 (18.0%)	1,512 (29.8%)	1,358 (26.8%)	1,108 (21.9%)	5,071 (100%)
EAMF	14 (10.1%)	3 (2.2%)	22 (15.9%)	19 (13.8%)	20 (14.5%)	34 (24.6%)	26 (18.9%)	138 (100%)
Drowning	6 (21.4%)	3 (10.7%)	3 (10.7%)	6 (21.4%)	0 (0.0%)	1 (3.6%)	9 (32.2%)	28 (100%)
Other	10 (14.5%)	13 (18.9%)	11 (15.9%)	8 (11.7%)	1 (1.4%)	1 (1.4%)	25 (36.2%)	69 (100%)

EIMF, exposure to inanimate mechanical forces; EAMF, exposure to animate mechanical forces.

### External causes of unintentional injuries

The leading cause of unintentional injury was foreign body (8,414 cases, 41.7%), followed by EIMF (5,071 cases, 25.1%), falls (4,458 cases, 22.1%), transport accidents (1,017 cases, 5.0%) and poisoning (723 cases, 3.6%) (Table 2). The external causes of unintentional injuries varied by age group. For children aged 0–1 year, the top three external causes were foreign body, falls and transport accidents. For children aged 1–10 years, they were foreign body, EIMF and falls. Lastly, for children aged 11–18 years, the primary causes were poisoning, EIMF and foreign body (Table 3).

**Table 2:** The variation in the ranking of causes of unintentional injuries from 2017–2023.

	Total (%)	2017	2018	2019	2020	2021	2022	2023
No. 1	Foreign body (41.7%)	Foreign body	Foreign body	Foreign body	Foreign body	EIMF	EIMF	EIMF
No. 2	EIMF (25.1%)	Falls	Falls	Falls	EIMF	Foreign body	Foreign body	Falls
No. 3	Falls (22.1%)	Poisoning	Transport accidents	EIMF	Falls	Falls	Falls	Foreign body
No. 4	Transport accidents (5.0%)	Transport accidents	Poisoning	Transport accidents	Transport accidents	Transport accidents	Transport accidents	Transport accidents
No. 5	Poisoning (3.6%)	Burn and scald	Burn and scald	Poisoning	Poisoning	Poisoning	Poisoning	Poisoning

**Table 3:** The distribution of causes of unintentional injuries by age, gender, and urban-rural location in central China, 2017–2023.^a^

Causes of injuries	Age						Sex		Location	
	<1 year	1–<4 years	4–<7 years	7–<11 years	11–18 years	Total	Male	Female	Urban	Rural
Foreign body	590 (41.4)	5,986 (52.1)	1,204 (25.8)	598 (25.8)	36 (13,9)	8,414 (41.7)	5,438 (41.6)	2,976 (41.9)	3,255 (31.6)	5,159 (52.4)
Falls	558 (39.2)	1,842 (16.0)	1,292 (27.7)	706 (30.4)	60 (23.2)	4,458 (22.1)	2,821 (21.6)	1,637 (23.2)	2,365 (23.0)	2,093 (21.2)
Transport accidents	90 (6.3)	424 (3.7)	298 (6.4)	171 (7.4)	31 (12.0)	1,017 (5.0)	652 (5.0)	365 (5.1)	445 (4.3)	572 (5.8)
Poisoning	36 (2.5)	472 (4.1)	92 (1.9)	66 (2.8)	57 (22.0)	723 (3.6)	418 (3.2)	305 (4.3)	394 (3.8)	329 (3.3)
Burn and scald	39 (2.7)	181 (1.6)	21 (0.5)	6 (0.3)	1 (0.3)	248 (1.3)	153 (1.2)	95 (1.3)	118 (1.1)	130 (1.3)
EIMF	81 (5.7)	2,534 (22.0)	1,708 (36.6)	689 (29.7)	59 (22.8)	5,071 (25.1)	3,417 (26.2)	1,654 (23.3)	3,628 (35.2)	1,443 (14.6)
EAMF	13 (0.9)	19 (0.2)	36 (0.8)	61 (2.6)	9 (3.5)	138 (0.7)	105 (0.8)	33 (0.5)	62 (0.6)	76 (0.8)
Drowning	0 (0)	15 (0.1)	5 (0.1)	6 (0.3)	2 (0.8)	28 (0.2)	16 (0.1)	12 (0.1)	8 (0.1)	20 (0.2)
Other	18 (1.3)	24 (0.2)	7 (0.2)	16 (0.7)	4 (1.5)	69 (0.3)	49 (0.3)	20 (0.3)	30 (0.3)	39 (0.4)
Total	1,425 (100)	11,497 (100)	4,663 (100)	2,322 (100)	259 (100)	20,166 (100)	13,069 (100)	7,097 (100)	10,305 (100)	9,861 (100)

^a^ Data represent as numbers (percentiles).

Foreign body was the most common external cause of unintentional injury. Among them, respiratory tract and gastrointestinal foreign body were the most frequent (4,619 cases [54.9%] and 2,869 cases [34.1%], respectively), while those in the nasal cavity, ear canal and vaginal were rare (387 cases [4.6%], 353 cases [4.2%] and 168 cases [2.0%] respectively). Respiratory tract, gastrointestinal and nasal cavity foreign body mainly occurred in children aged 1–3 years, while ear canal and vaginal foreign body mostly occurred in children aged 4–6 years. In terms of seasonal patterns for respiratory foreign bodies, 1,792 cases (38.8%) occurred during Spring (January to March), 901 cases (19.5%) during Summer (April to June), 868 cases (18.8%) during Autumn (July to September), and 1,058 cases (22.9%) during Winter (October to December).

EIMF was the second most common external cause of unintentional injury. It mainly occurred in children aged 1–6 years, with a male-to-female ratio of 2.1:1. EIMF included various causes, of which the most common was being struck by or against an object (4,534 cases, 89.4%), followed by being caught, crushed, rolled over or pinched by an object (230 cases, 4.5%).

Falls were the third most common external cause of unintentional injury. It mainly occurred in children aged 0–6 years, with a male-to-female ratio of 1.7:1.

### Trends of unintentional injuries from 2017–2023

**Figure 1:**
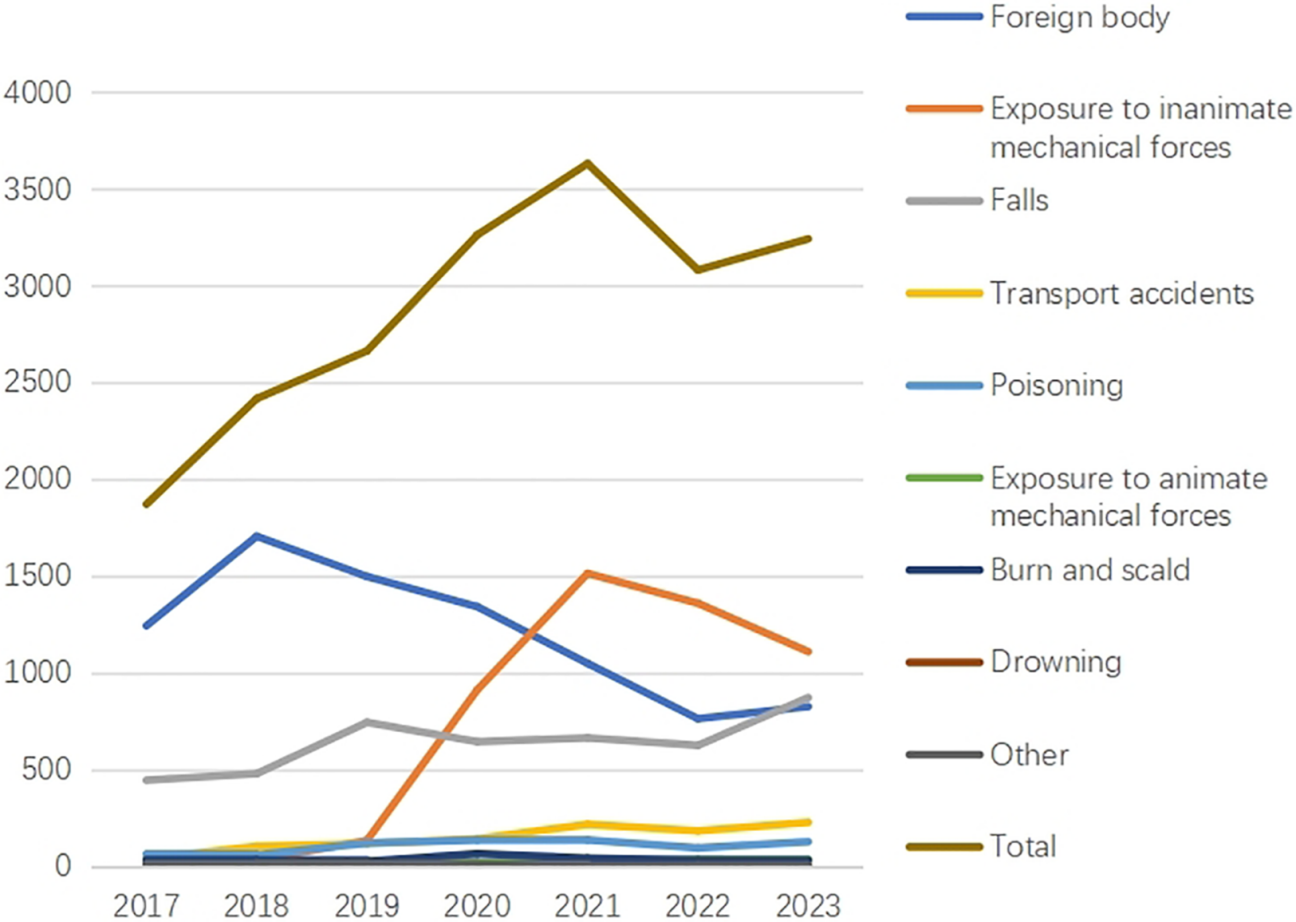
Distribution of different causes of unintentional injuries in central China between 2017 and 2023.

Overall, there were statistically significant differences in the age, gender, urban-rural distribution, and causes of injuries among unintentional injury patients across different years (*p* < 0.001 for each). Notably, patient age showed a gradual increase from 2017–2023. In 2019 and 2020, the proportion of male patients was significantly higher than in other years (*p* < 0.001 for each). Prior to 2020, unintentional injuries were more common in rural areas than in urban areas; however, this trend reversed after 2020. The total number of unintentional injuries exhibited an increasing trend from 2017–2022, followed by a decrease from 2022–2023 (Figure 1). Regarding external causes of injuries, foreign body showed a decreasing trend after 2018, while EIMF demonstrated an increasing trend from 2017–2021 (Table 1).

### Body parts affected by unintentional injuries

The most frequently injured body parts by unintentional injuries were head (7,789 cases, 71.3%), shoulder and upper arm (796 cases, 7.3%), and wrist and hand (427 cases, 3.9%) (Table 4).

**Table 4:** Body parts affected by different causes of unintentional injuries.^a^

Causes of injuries	S00–S09	S10–S19	S20–S29	S30–S39	S40–S49	S50–S59	S60–S69	S70–S79	S80–S89	S90–S99	T00–T07
Falls	2,814 (63.1)	18 (0.4)	28 (0.6)	140 (3.1)	702 (15.7)	327 (7.3)	45 (1.0)	129 (2.9)	102 (2.3)	23 (0.5)	130 (2.9)
Transport accidents	480 (47.2)	5 (0.5)	96 (9.4)	136 (13.4)	74 (7.3)	43 (4.2)	24 (2.4)	47 (4.6)	47 (4.6)	38 (3.7)	27 (2.7)
EIMF	4,388 (86.5)	9 (0.2)	6 (0.1)	62 (0.3)	5 (6.9)	17 (0.5)	351 (0.8)	23 (0.8)	41 (0.8)	59 (1.2)	110 (2.2)
EAMF	60 (43.5)	7 (5.1)	2 (1.4)	36 (26.1)	3 (2.2)	2 (1.4)	3 (2.2)	8 (5.8)	0 (0)	1 (0.7)	16 (11.6)
Burn and scald	47 (18.9)	27 (10.9)	22 (8.9)	15 (6.0)	12 (4.8)	10 (4.0)	4 (1.7)	45 (18.1)	0 (0)	4 (1.7)	62 (25.0)
Total	7,789 (71.3)	66 (0.6)	154 (1.4)	389 (3.5)	796 (7.3)	399 (3.6)	427 (3.9)	252 (2.3)	190 (1.8)	125 (1.1)	345 (3.2)

^a^ Data represent as numbers (percentiles).

S00–09: injuries to the head; S10–19: injuries to the neck; S20–29: injuries to the thorax; S30–39: injuries to the abdomen, lower back, lumbar spine and pelvis; S40–49: injuries to the shoulder and upper arm; S50–59: injuries to the elbow and forearm; S60–69: injuries to the wrist and hand; S70–79: injuries to the hip and hand; S80–89: injuries to the knee and lower leg; S90–99: injuries to the ankle and foot; T00–07: injuries involving multiple body regions.

### Hospitalization costs and length of stay

The total hospitalization cost for unintentional injuries was 20,810,870.4 USD, median cost of 758.7 (556.4, 1,186.2) USD. The top three external causes in terms of total cost were foreign body (7,417,598.9 USD), falls (5,677,921.5 USD) and EIMF (3,975,202.3 USD). The highest median cost was for drowning (1,542.3 USD), followed by transport accidents (1,287.2 USD). The longest average length of stay was for transport accidents (15 days) (Table 5).

**Table 5:** The hospitalization cost of different causes of unintentional injuries.

Causes of injuries	Total cost, $	Individual cost, $	Individual hospital stay, day
Foreign body	7,417,598.9	837.3 (557.9, 1,657.4)	3 (1, 4)
Falls	5,677,921.5	837.4 (557.9, 1,658.8)	5 (2, 9)
Transport accidents	2,524,520.8	1,287.2 (721.4, 2,482.3)	9 (5, 18)
Poisoning	635,022.4	462.3 (269.3, 918.4)	3 (2, 6)
Burn and scald	232,973.5	738.3 (396.1, 1,219.1)	7 (4, 11)
EIMF	3,975,202.3	707.3 (621.8, 788.7)	1 (1, 1)
EAMF	185,556.2	715.7 (477.7, 1,073.0)	5 (1, 10)
Drowning	75,166.2	1,542.3 (580.9, 3,026.7)	6.5 (3.75, 11.25)
Other	86,908.6	887.4 (555.3, 2,037.9)	7 (4, 12)
Total	20,810,870.4	758.7 (556.4, 1,186.2)	2 (1, 5)

## Discussion

To the best of our knowledge, this is the first and largest study to describe the hospitalized unintentional injuries among children in Central China from 2017–2023. Our study revealed a significant gender difference, with a male-to-female ratio of 1.8:1. This finding is in line with previous reports from various regions of the world (15–18). The most vulnerable age group for unintentional injuries was 1–6 years old; this age group also exhibited a similar trend in both national and international studies (19, 20). We speculated that this might be attributed to the higher activity level, stronger curiosity, and lower safety awareness of children under 6 years old (21). There was no significant difference between urban and rural areas in the distribution of unintentional injuries; however, rural areas had a slightly higher incidence rate than urban areas. This might be associated with the lower educational attainment of caregivers in rural areas and their lower awareness of preventing unintentional injuries (22). Nevertheless, there were divergent factors between urban and rural areas regarding the external causes of unintentional injuries. In our study, rural areas suffered more injuries caused by foreign bodies, traffic accidents, and drowning than urban areas; while urban areas encountered more injuries caused by exposure to EIMF, falls, and poisoning than rural areas. Interestingly, the urban-rural distribution reversed in our study after 2020. This might be related to the outbreak of COVID-19 in China at the end of 2019 (23). According to the control policy at that time, children with injuries were treated locally in rural areas; thus our hospital's referral number decreased significantly (24).

In our study, we discovered that foreign body was the leading cause of unintentional injury in Central China; however, this finding diverged from previous reports both domestically and internationally. A cross-sectional survey from Egypt (25), a cohort study from Brazil (26) and a large-scale household survey involving more than one million people from Bangladesh (27) all indicated that falls were the predominant external cause of unintentional injury (13). Likewise, studies conducted in northwestern, southern, and Shanghai regions in China corroborated this finding (14, 20). The possible reasons for this discrepancy are as follows: First, Central China, especially Henan Province, is a major agricultural province, producing a variety of nuts, which increases the risk of respiratory tract foreign bodies among children. Second, we observed that the incidents of respiratory tract foreign body reached their peak during springtime (January to March), coinciding with the traditional Chinese Spring Festival. According to the custom of preparing nuts for the festival, the exposure to unintentional injury among children increased. About 75% of respiratory tract foreign bodies were diagnosed by parents who provided clear histories of aspiration, and another 25% were diagnosed due to persistent pneumonia. Studies have shown that by raising caregivers’ safety awareness and providing timely diagnosis and treatment, the incidence and mortality rates of respiratory tract foreign body can be significantly reduced (28, 29). Therefore, we recommend that education regarding foreign bodies be further strengthened. This includes emphasizing the importance of providing age-appropriate foods for young children, particularly in rural areas in Central China.

Gastrointestinal foreign bodies can occur at any age, but in our study, 68.3% of them occurred in children under 3 years of age, and 92.2% of them occurred in children under 6 years of age, which was in agreement with previous reports from Hong Kong and South Africa (30–34). The reason might be related to the lack of self-protection awareness among children in this age group who are curious about external objects and their own oral cavities (35). The high incidence of children under 3 years of age might also be related to the incomplete development of chewing ability, immature swallowing function, and inadequate ability to distinguish between edible and inedible items (36). In our study, we found that although foreign bodies were the most common cause of unintentional injury in Central China, the incidence had shown a gradual decline after 2018, due to comprehensive and continuous social education. Therefore, foreign bodies are preventable and manageable.

The second leading external cause of unintentional injury in Central China was EIMF, which was influenced by various factors, such as socio-economic status, environmental conditions, population characteristics, and behavioral habits (37). In our study, EIMF was more prevalent among children aged 1–6 years, and more frequent in urban areas. The possible reasons for this were the industrialization and urbanization processes in China, which resulted in the increased use of mechanical devices, as well as the complex interpersonal relationships and social conflicts in cities, which amplified the sources and frequency of mechanical forces. This demanded our attention and further corresponding prevention and control measures.

Falls were the third leading external cause of unintentional injury in Central China. Falls could result in head and facial injuries, traumatic spinal cord injuries and cervical spine injuries in infants and young children (38, 39), and falls were also one of the main causes of death among children. The incidence of falls among children was affected by various factors, such as age, gender, health status, environmental conditions, behavioral habits (39). A project conducted at Arkansas Children's Hospital demonstrated that implementing evidence-based interventions for children, such as providing falls prevention education to patients and families, helped lower patient falls (40). Therefore, it is crucial to enhance public education on fall prevention and improve pediatric care to reduce the incidence of falls. Additionally, emphasizing child-friendly home environment modifications and encouraging engaging activities that promote brain development and balance training is essential. Examples of such activities include yoga ball exercises, knee balance exercises, closed-eye exercises on balance tables, and one-legged standing, etc.

A global burden of disease study for children in 188 countries in 2013 reported that road injuries were the primary cause of death for children aged 5–9 years in North America, Latin America and the Caribbean, and Oceania, while drowning was the most common cause of death in most Eastern European, East Asian, and Southeast Asian countries (41). Drowning was closely associated with temperature, environmental risks, and water-related risk activities, and it occurred more frequently in rural areas with abundant water resources (42). There were fewer drowning patients in our study, and the possible reasons were that Central China is located in a plain area with few lakes and swamps. Moreover, the Chinese government emphasizes the public education on drowning prevention, for example, the safety education platform for primary and secondary schools in China includes drowning protection, which aims to educate students, parents, and teachers to reduce the occurrence of drowning. Therefore, it is very important to strengthen the publicity and education on unintentional injuries.

However, our study has some limitations, such as the lack of data on the severity and outcomes of unintentional injuries, and the risk factors associated with unintentional injuries, such as the physical and mental health status of children, family income and parents' education level. Future research should address these issues and investigate the underlying mechanisms and determinants of unintentional injuries among children, to guide the development of relevant policies to prevent these injuries. Since there were no electronic records for outpatient cases prior to 2020, our analysis of potential trends in unintentional injuries is limited to the data from hospitalized patients during the study period and does not include outpatient cases.

In conclusion, we analyzed the unintentional injuries among hospitalized children aged 0–16 years from 2017–2023 in Central China. Firstly, we found that unintentional injuries imposed a heavy burden on society and families, in terms of the total number of cases, length of hospital stay, and hospital expenses. However, the number of cases and the urban-rural distribution showed significant trend changes from 2017–2023. Secondly, the most common external causes were foreign bodies, EIMF and falls. Moreover, the external causes varied by age group, gender, and region. Finally, we found that unintentional injuries among children were preventable and manageable. Our study provides valuable insights into the epidemiology and economic impact of unintentional injuries among children in Central China, and indicates the need for more effective prevention and control measures, especially for EIMF.

## Data Availability

The original contributions presented in the study are included in the article, further inquiries can be directed to the corresponding author.
